# Plasma Metabolomic Profiles Reflective of Glucose Homeostasis in Non-Diabetic and Type 2 Diabetic Obese African-American Women

**DOI:** 10.1371/journal.pone.0015234

**Published:** 2010-12-10

**Authors:** Oliver Fiehn, W. Timothy Garvey, John W. Newman, Kerry H. Lok, Charles L. Hoppel, Sean H. Adams

**Affiliations:** 1 Genome Center, University of California Davis, Davis, California, United States of America; 2 Department of Nutrition Sciences, University of Alabama at Birmingham and the Birmingham VA Medical Center, Birmingham, Alabama, United States of America; 3 Obesity and Metabolism Research Unit, USDA-Agricultural Research Service Western Human Nutrition Research Center, University of California Davis, Davis, California, United States of America; 4 Department of Nutrition, University of California Davis, Davis, California, United States of America; 5 Department of Pharmacology, Case Western Reserve University, Cleveland, Ohio, United States of America; Pennington Biomedical Research Center, United States of America

## Abstract

Insulin resistance progressing to type 2 diabetes mellitus (T2DM) is marked by a broad perturbation of macronutrient intermediary metabolism. Understanding the biochemical networks that underlie metabolic homeostasis and how they associate with insulin action will help unravel diabetes etiology and should foster discovery of new biomarkers of disease risk and severity. We examined differences in plasma concentrations of >350 metabolites in fasted obese T2DM vs. obese non-diabetic African-American women, and utilized principal components analysis to identify 158 metabolite components that strongly correlated with fasting HbA1c over a broad range of the latter (r = −0.631; p<0.0001). In addition to many unidentified small molecules, specific metabolites that were increased significantly in T2DM subjects included certain amino acids and their derivatives (i.e., leucine, 2-ketoisocaproate, valine, cystine, histidine), 2-hydroxybutanoate, long-chain fatty acids, and carbohydrate derivatives. Leucine and valine concentrations rose with increasing HbA1c, and significantly correlated with plasma acetylcarnitine concentrations. It is hypothesized that this reflects a close link between abnormalities in glucose homeostasis, amino acid catabolism, and efficiency of fuel combustion in the tricarboxylic acid (TCA) cycle. It is speculated that a mechanism for potential TCA cycle inefficiency concurrent with insulin resistance is “anaplerotic stress” emanating from reduced amino acid-derived carbon flux to TCA cycle intermediates, which if coupled to perturbation in cataplerosis would lead to net reduction in TCA cycle capacity relative to fuel delivery.

## Introduction

Type 2 diabetes mellitus (T2DM) is a progressive disease in which increasingly poor insulin sensitivity and hyperinsulinemia precede, typically for several years, the onset of frank diabetes [1]. The central feature of the insulin resistance involves reduced insulin-mediated glucose uptake and metabolism, particularly in skeletal muscle. However, evidence also suggests dysregulated fatty acid metabolism and tissue lipid accumulation as being associated with the development of insulin resistance and T2DM [2]; [3]. Furthermore, in human obesity and in some obese, diabetic animal models an elevation of circulating branched chain amino acid (BCAA) concentrations has been reported [4]–[9]. Thus, insulin resistance progressing to T2DM is marked by a broad perturbation of macronutrient intermediary metabolism.

Excessive cellular accumulation of lipids in muscle [10]–[17], liver [18], and adipocytes [19] is associated with insulin resistance in those tissues. Lipotoxicity in pancreatic β-cells also is proposed as a factor leading to loss of β-cell function in T2DM [20]. In skeletal muscle specifically, lower long-chain fatty acid (LCFA) oxidation in the fasted state and a blunted increase of carbohydrate oxidation in response to increased insulinemia (“metabolic inflexibility”) is a common feature of pre-diabetes and T2DM [21]. Insulin resistance may be accompanied by a greater mismatch between muscle LCFA delivery and tissue oxidative capacity, leading to accumulation of by-products of incomplete LCFA oxidative catabolism [22]. Consistent with this model, acylcarnitines resulting from incomplete LCFA β-oxidation were more prevalent and elevated in the plasma of type 2 diabetic women compared to non-diabetics [23]. However, the tissue source of these metabolites cannot be pinpointed from plasma patterns alone. The molecular factors linking inefficient LCFA catabolism to impaired insulin action remain controversial, but may include build-up of cytosolic ceramide and diacylglyerol (DAG) that inhibit Akt/PKB and activate PKC enzymes, respectively (see reviews by [24]–[26]). Increased tissue exposure to saturated fatty acids can activate pro-inflammatory cascades associated with insulin resistance via toll like receptors 2 and 4, and we reported that at least some acylcarnitines elevated in T2DM plasma can trigger NFκB-driven gene expression, suggesting that these metabolites have pro-inflammatory properties as well [23].

Understanding the biochemical networks underlying metabolic homeostasis and their association with insulin sensitivity will help to clarify diabetes etiology, and should foster the discovery of new biomarkers of disease risk and severity. It would be particularly useful to identify metabolite signatures specific to muscle LCFA oxidation considering the importance of lipid metabolism in this tissue to whole-body insulin sensitivity. However, there is a paucity of comparative experimental models in which β-oxidation is altered *exclusively* or *predominantly* in muscle cells. To address these problems, we have applied metabolomics platforms to compare plasma metabolite patterns in weight-matched obese non-diabetic and T2DM African-American women, with or without an uncoupling protein 3 (UCP3) g/a missense polymorphism that results in substantial reductions in whole-body LCFA oxidation [27]. Considering that UCP3 is essentially muscle-specific in humans, examination of metabolic patterns in persons harboring the UCP3 g/a polymorphism holds promise to uncover muscle-specific moieties reflective of altered muscle β-oxidation. The archived plasma samples examined herein are identical to those used in our recent report of acylcarnitine profiles in this cohort [23], and this complementary work extends the metabolite coverage to >400. The results indicate that variances in a unique subset of metabolites successfully discriminate non-diabetics from T2DM subjects. The metabolite patterns highlight that indices of poor blood sugar control, and markers of inefficient TCA cycle function, strongly correlate with increased plasma BCAA concentrations, which we propose reflects disruption of normal amino acid catabolism and hence imbalanced anaplerosis. The data also point to a potential role of mitochondrial UCP3 in regulation of glutamate/α-ketoglutarate/butanoyl-CoA metabolism.

## Methods

### Human Volunteers and Blood Plasma Collection

Comprehensive details regarding the study cohort and sample collection were previously-described [23]. Briefly, archived plasma samples derived from body mass index- (BMI) and age-matched overweight to obese type 2 diabetic (n = 44) and non-diabetic (n = 12) Gullah-speaking African-American women with or without a UCP3 g/a missense polymorphism were evaluated. Volunteers were recruited as part of the Project SuGAR study described in detail elsewhere [28]–[31]. Considering that this group is of a single sex, displays an extraordinarly low genetic admixture, lives in a relatively small geographical space, and has a common dietary intake pattern, we anticipate that the cohort is well-suited for metabolomics studies since biological metabolite signal-to-noise should be low. Ethics Statement: Studies were approved by the Institutional Review Boards of the Medical University of South Carolina, University of Alabama at Birmingham, and the University of California, Davis, and all participants provided written informed consent. Blood was collected by arm venipuncture between ∼08:00–09:00 into EDTA-treated collection tubes after an overnight fast (no food or drink since 20:00 the night before). Plasma was frozen at −20°C for 1–7 days before transport to −80°C freezers for longer-term storage. Volunteers were asked to avoid unusual activity and intentional exercise in the 3 days leading up to the study, and were instructed to continue to eat their habitual diet without unusual deviations. Patients with T2DM did not take doses of oral agents on the evening before and on the morning of study. Patients treated with insulin could take regular or rapid acting insulin at dinner the night before the study but were instructed to withhold any intermediate- or long-acting insulin on the evening before, and to avoid insulin injections on the morning of the study.

### Metabolite Analysis

Plasma samples for metabolomics assays were thawed on ice, aliquoted, re-frozen on dry ice, and stored at −80°C prior to delivery to the Fiehn lab. Plasma aliquots (15 µL) were extracted and derivatized as reported previously [29] using 1 mL of degassed acetonitrile:isopropanol:water (3∶3∶2; v/v/v) at −20°C, centrifuged and decanted with subsequent evaporation of the solvent to complete dryness. A clean-up step with 500 µL acetonitrile/water (1∶1; v/v) removed membrane lipids and triglycerides and the supernatant was dried down again. A set of 13 C8–C30 fatty acid methyl ester internal standards were added and samples were derivatized by 10 µL methoxyamine hydrochloride in pyridine followed by 90 µl MSTFA (1 mL bottles, Sigma-Aldrich) for trimethylsilylation of acidic protons. A Gerstel MPS2 automatic liner exhange system (Mülheim an der Ruhr, Germany) was used to inject 0.5 µL of sample at 50°C (ramped by to 250°C) in splitless mode with 25 s splitless time. Analytes were separated using an Agilent 6890 gas chromatograph (Santa Clara, CA) equipped with a 30 m long, 0.25 mm i.d. Rtx5Sil-MS column with 0.25 µm 5% diphenyl film and additional 10 m integrated guard column (Restek, Bellefonte PA). Chromatography was performed with constant flow of 1 mL/min while ramping the oven temperature from 50°C for to 330°C with 22 min total run time. Mass spectrometry was done by a Leco Pegasus IV time of flight mass spectrometer (St. Joseph, MI) with 280°C transfer line temperature, electron ionization at −70eV and an ion source temperature of 250°C. Mass spectra were acquired from m/z 85–500 at 17 spectra s^−1^ and 1850 V detector voltage. Result files were exported to our servers and further processed by our metabolomics BinBase database [32]. All database entries in BinBase were matched against the Fiehn mass spectral library of 1,200 authentic metabolite spectra using retention index and mass spectrum information or the NIST05 commercial library. Identified metabolites were reported if present within at least 50% of the samples per study design group (as defined in the SetupX database) [33]. Peak heights of quantifier ions defined for each metabolite in BinBase were normalized to the sum intensities of all known metabolites and used for statistical investigation. External 5-point calibration curves established with quality control mixtures containing 30 metabolites controlled for instrument sensitivity. Each chromatogram was further controlled with respect to the total number of identified metabolites and total peak intensities to ensure that outliers did not confound the subsequent statistical analysis.

### Statistical Analyses

A mixture of univariate and multivariate statistics were applied to the investigation of changes in this study. Differences in mean plasma analyte concentrations between subjects with different UCP3 genotypes or diabetic status were initially evaluated using unpaired Student's t-tests with multiple comparison adjustments made using the method of Benjamini and Hochberg [34]. A false discovery rate (FDR) of 20% (i.e., q = 0.2) was applied. Principal components analyses (PCA) were used to independently assess metabolites that segregated with genotype and diabetic status. Specifically, all detected metabolites were used as input variables, and principal components were ranked on significant differences between scores of sample classifications. The PCA was performed using the Microsoft Excel add-in developed by the Bristol Centre for Chemometrics, University of Bristol, UK (http://www.chm.bris.ac.uk/org/chemometrics/chemometrics.html). The Pearson's correlation statistic was used to determine relationships between select metabolites (PrismGraph, GraphPad, San Diego, CA). All results are presented as means ± SEM, and P<0.05 was considered statistically significant.

## Results

Over 700 discrete signals were detected using GC-TOF mass spectrometry for each plasma sample. After applying the BinBase database filtering, 366 metabolites passed stringent analytical quality control measures, and used for comparisons of diabetic vs. non-diabetic and UCP3 g/g vs. UCP3 g/a plasma metabolomic profiling. The identities of these metabolites are provided in **Table S1** (genotype comparisons) and **Table S2** (diabetes comparisons). Metabolites lacking full structural identification (“unknowns”) are unambiguously described by BinBase (BB) numbers and full mass spectra, quantifier ions and retention indices. These data are publically available and queryable against all 24,000 samples in BinBase (http://eros.fiehnlab.ucdavis.edu:8080/binbase-compound/).

### Plasma Metabolite Profiles in Non-Diabetic UCP3 g/g and g/a Polymorphs

In non-diabetic persons harboring the missense g/a UCP3 allele, although there were 35 metabolites with mean plasma concentrations at least 50% higher vs. g/g subjects (**Table S1**), the difference in only 2 metabolites achieved statistical significance assessed by unpaired two-tailed t-tests (BB226860 and BB219174; **Table 1**). In contrast, 14 of 18 plasma metabolites reduced by at least 50% in g/a polymorphs were significantly different compared to g/g subjects (**Table 1**). However, changes did not achieve statistical significance after applying a 20% FDR correction (see Methods). Regardless, it is notable that concentrations of 2-oxoglutarate (α-ketoglutarate) and glutamate were both reduced in non-diabetic subjects harboring the g/a allele. In addition, our previous report of a 36% reduction in plasma lactate concentration in non-diabetic g/a subjects measured by standard clinical chemistry analyses [23] was confirmed by mass spectral analysis (**Table S1**). Most metabolites that were significantly altered in non-diabetic g/a individuals vs. g/g individuals remain unidentified (**Table 1** and **Table S1**).

**Table 1:** Plasma metabolites with significantly-altered concentrations in non-diabetic obese African-American women harboring a UCP3 g/a missense allele.

	g/g genotype (n = 6)	g/a genotype (n = 6)	Relevant Metabolic Pathway	g/a to g/g Ratio
**Increased in non-diabetic g/a:**				
BB226860	2552±669	5851±1302	unknown	2.29*
BB219174	6966±988	10253±1074	unknown	1.47*
**Decreased in non-diabetic g/a:**				
BB223521	3569±340	2516±318	unknown	0.71*
phosphoric acid	1540725±106083	1065028±88171	acid/base balance?	0.69**
BB223506	4884±343	3288±483	unknown	0.67*
BB281189	432579±45901	285112±14032	unknown	0.66**
inulobiose	1223±152	795±87	carbohydrate	0.65*
BB228147	579±67	374±49	unknown	0.65*
BB211382	36484±3617	21651±3493	unknown	0.59**
cysteine	24519±4380	13827±1928	amino acid	0.56*
2-oxoglutarate (α-ketoglutarate)	2209±240	1182±130	TCA cycle/transamination	0.54**
BB281112	18342±2886	9189±2265	unknown	0.50*
BB228144	3874±659	1910±524	unknown	0.49*
BB239966	2358±418	1158±308	unknown	0.49*
glutamic acid (glutamate)	48146±7473	21048±1427	amino acid	0.44**
BB222169	25526±4397	10743±4133	unknown	0.42*

Values are quantifier peak height means ± SEM; see Supplemental Table S1 for full list of metabolites including those whose concentration differences were not statistically significant;

*p≤0.05;

**p≤0.01 (unpaired t-test).

Note that after application of a 20% false discovery rate (see Methods) these differences did not achieve statistical significance.

### Plasma Metabolite Comparisons in Non-Diabetic and Type 2 Diabetic Subjects

As expected, comprehensive metabolomics analysis of diabetic vs. non-diabetic plasma revealed significantly increased concentrations of glucose, long-chain fatty acids (LCFAs: oleic, palmitoleic, palmitic), and the ketone body 3-hydroxybutanoic acid (β-hydroxybutyrate) in T2DM subjects (**Table 2**). The mean plasma concentrations of 36 metabolites increased ≥50% in T2DM subjects vs. non-diabetics (**Table S2**). A total of 59 plasma metabolites were decreased in T2DM subjects: 18 metabolites were reduced by at least 25% in diabetics vs. non-diabetics, and 1 of these had a concentration >50% lower in T2DM (BB281134; **Table S2**). All metabolites that were significantly different when comparing diabetic vs. non-diabetic subjects passed the 20% FDR threshold. Most metabolites altered in diabetics remain unidentified in terms of chemical nomenclature; known metabolites are listed in **Table 2**.

**Table 2:** Identifiable plasma metabolites with significantly-altered concentrations in obese non-diabetic vs. type 2 diabetic African-American women.

	Non-Diabetic (n = 12)	Diabetic (n = 43)	Relevant Metabolic Pathway	Diabetic/Non-Diabetic Ratio
**Increased in Diabetes:**				
3-hydroxybutanoic acid (β-hydroxybutryrate)	10676±1455	47424±9450	lipid/fatty acid	4.44*
oleic acid	8837±1105	23377±2189	lipid/fatty acid	2.65***
gluconic acid	2570±230	5317±229	carbohydrate	2.07****
fructose	255053±34001	517922±18549	carbohydrate	2.03****
palmitoleic acid	6286±1444	11400±1018	lipid/fatty acid	1.81*
3,6-anhydrogalactose	1660±147	2920±116	carbohydrate (microbial?)	1.76****
glucuronic acid	1718±151	2844±264	carbohydrate	1.66*
glucose	1057532±90953	1644213±56650	carbohydrate	1.56****
heptadecanoic acid	11630±554	17911±1564	lipid/fatty acid	1.54*
inulobiose	1009±106	1546±92	carbohydrate	1.53**
leucine	110271±14147	164281±9806	amino acid	1.49**
2-hydroxybutanoic acid (α-hydroxybutryrate)	100560±21376	146853±9844	amino acid	1.46*
2-deoxyerythritol	8270±727	10950±383	lipid/fatty alcohol	1.32**
palmitic acid	75185±5308	98294±6003	lipid/fatty acid	1.31*
2-ketoisocaproic acid (α-ketoisocaproate)	4809±462	6169±309	amino acid	1.28*
uridine	850±51	1085±51	pyrimidine	1.28*
cystine	30534±3583	38496±1818	amino acid	1.26*
xylose	4388±290	5479±221	carbohydrate/pentose phosphate	1.25*
histidine	44969±2332	56071±2178	amino acid	1.25**
stearic acid	598153±31201	719217±24664	lipid/fatty acid	1.20*
**Decreased in Diabetes:**				
benzylalcohol	17762±1062	15741±405	phenolic metabolite or xylene (microbial?)	0.89*
benzoic acid	37841±2445	32968±1066	phenolic metabolite or xylene (microbial?)	0.88*
lysine	170439±13635	141626±6008	amino acid	0.83*
ethanolamine	479789±42252	380214±19511	choline precursor	0.79*
arachidonic acid	35123±3669	26058±1410	lipid/fatty acid	0.74**
glycine	326074±41720	239650±16035	amino acid	0.74*
glycerol-3-phosphate (glycerol-α-phosphate)	23920±2430	16571±1018	glycerophospholipid	0.69**

Values are quantifier peak height means ± SEM; see Supplemental Table S2 for information on unknown metabolites significantly changed in T2DM;

*p≤0.05;

**p≤0.01;

***p≤0.001;

****p≤0.0001 (unpaired t-test).

**Figure 1:**
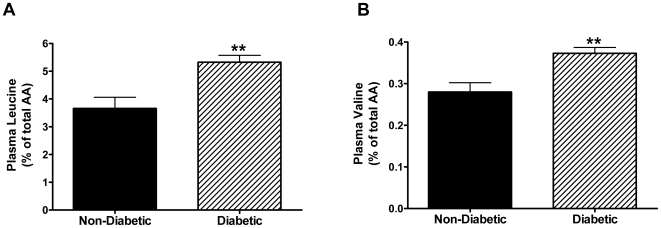
Relative plasma concentrations of leucine (A) and valine (B) are increased in type 2 diabetic African-American obese women. Results are expressed as total quantifier peak height percent of all summed plasma amino acid (AA) peak heights, i.e., as % of total AA. Bars represent the mean ± SEM for n = 12 and n = 43 non-diabetic and diabetic subjects, respectively. **p<0.01, unpaired t-test. Absolute quantifier peak heights are presented in the Results.

**Figure 2:**
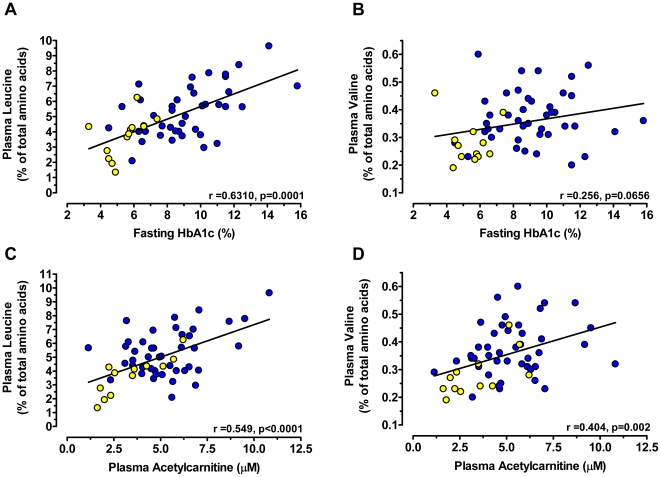
Plasma branched chain amino acid (BCAA) concentrations are correlated with fasting HbA1c% and plasma acylcarnitine concentrations in non-obese (yellow circles) and type 2 diabetic (blue circles) obese women. Shown are correlations between plasma leucine (**A**,**C**) and valine (**B**,**D**) enrichments (% of their concentrations relative to total measured amino acid concentrations; see Figure 1 legend) with fasting blood HbA1c (top panels) or plasma acetylcarnitine concentration (bottom panels). Pearson's r and p values for the correlations are given within the figures.

Of the metabolites that differed in T2DM subjects, it was notable that plasma leucine concentration was significantly increased by ∼50% (**Table 2**), and its initial catabolic metabolite, 2-ketoisocaproic acid (α-ketoisocaproate), was significantly increased by ∼27% (**Table 2**). Mean plasma valine concentration was ∼20% higher in type 2 diabetic subjects vs. non-diabetics (**Table S2**), but this difference was not statistically significant. When leucine and valine were each considered in terms of their enrichment in the total plasma amino acid pool (expressed as a % of the total summed quantifier ion peak heights of all detected amino acids), the diabetes-related increases in these BCAAs were even more apparent and statistically significant for both leucine % and valine % (**Figure 1**). Leucine % enrichment increased concurrent with worsening blood sugar control since degree of enrichment correlated significantly with hemoglobin A1c (HbA1c%)(**Figure 2A**). The relationship between valine % and HbA1c was less robust (**Figure 2B**).

**Figure 3:**
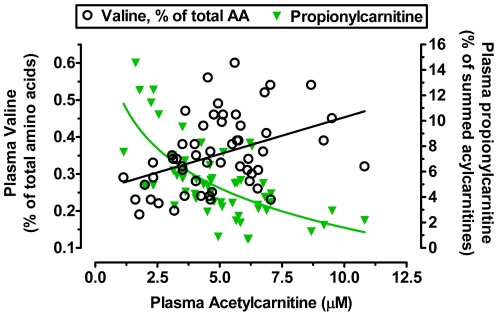
Relative plasma concentrations of propionylcarnitine (green symbols) were reduced concurrent with increases in the relative plasma concentrations of a precursor, valine, with increasing plasma acetylcarnitine concentration in obese African-American women. Symbols represent individuals included in metabolomics studies described in the text.

Using results from our previous study of plasma acylcarnitine patterns in these same samples [23], both leucine and valine % enrichment in the plasma amino acid pool were found to correlate strongly (p<0.001) with acetylcarnitine concentration (**Figure 2C and 2D**). It can also be seen that increasing valine % enrichment is coincident with a reduction in relative concentrations of propionylcarnitine, a marker of the valine catabolic product propionyl-CoA; these patterns are associated with increasing acetylcarnitine concentrations (**Figure 3**).

**Figure 4:**
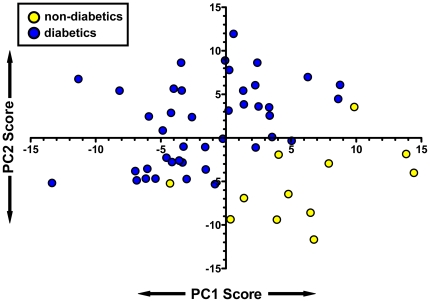
Separation of non-diabetics (yellow circles) from type 2 diabetics (blue circles) due to variance in plasma metabolite factors. Principal components analysis (PCA) in dimensions 1 and 2 using 158 metabolites illustrates differential distribution of diabetic and non-diabetic subjects along the PC1 (X axis) and PC2 (Y axis) axes, with each symbol plotting PC1-PC2 scores for a given subject. Metabolite components whose variance-derived loadings values contributed most to the PC separations scores are listed in Supplemental Table S3. Summarizing the loading contributions from known compounds, elevated fatty acids and the enrichment of the amino acid pool with branched chain AAs segregated diabetics from controls in PC1, while elevations In various carbohydrates as well as a suite of amino acids separated diabetics from controls in PC2.

**Figure 5:**
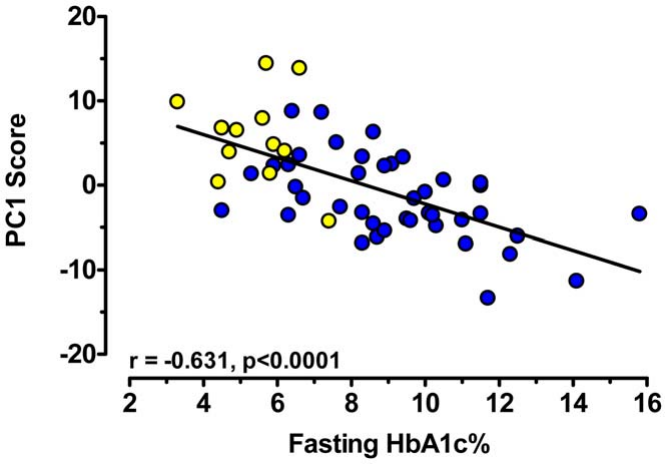
Correlation between PC1 scores and blood HbA1c% in non-diabetic (yellow circles) and type 2 diabetic (blue circles) obese women. PC1 scores derived from PCA analysis (see text and Figure 4 legend) were used for correlation to a marker of blood sugar control. Pearson's r and p values for the correlation is given within the figure.

To identify the specific plasma metabolites with distributions most affected by subjects' diabetes status, we first employed PCA, an unsupervised multivariate analysis approach, and considered four principal components (PC) dimensions. Metabolite variation in PC dimensions 2 and 3 (PC2, PC3) explained 16.8% of the variance between groups, and the differences between diabetic vs. non-diabetic PC scores were highly significant (P<0.001 for both PC2 and PC3)(data not shown). A principal components (metabolites) selection criterion for diabetic and non-diabetic group discrimination was set for components with loadings (i.e., variance contribution) ≥1 standard deviation from the mean loading value in each of the discriminating PC2 and PC3 dimensions. The 158 metabolite components emerging from this approach were used in a secondary PCA to further refine the principal components that most accurately predict the type 2 diabetic phenotype (this analysis did not include glucose since this metabolite defines diabetes status). In this secondary PCA, PC1 explained 40% of the group variance, and the mean PC1 scores in diabetics and non-diabetics were significantly different (p<0.0001), indicating successful phenotype discrimination. An additional 18.3% of the total group variance was accounted for in the PC2 dimension, and the mean PC2 scores in diabetics and non diabetics were significantly different (p<0.0001). The PC1 and PC2 scores were thus plotted for each of the diabetic and non-diabetic subjects to visualize the magnitude of separation of the groups (**Figure 4**): excellent separation of diabetic and non-diabetic subject cohorts was achieved. Loadings for each of the discriminating factors may be found in **Table S3**; metabolites with loadings at the highest and lowest extremes in PC1 and PC2 are those with variances most strongly impacting the phenotype separation in the PC1 and PC2 dimensions in Figure 4. The PC1 scores derived from the selected metabolites were highly correlated with the degree of blood sugar control (blood HbA1c%), indicating a significant association between person-to-person differences in these metabolites and the diabetic phenotype (**Figure 5**).

## Discussion

Type 2 diabetes mellitus is a disease of abnormal intermediary metabolism of an array of nutrients including glucose. For instance, the importance of lipid metabolism in diabetes was highlighted in the seminal studies by Randle et al. indicating that muscle glucose utilization can be inhibited by increased provision of long chain fatty acids (LCFAs)[35]. Later perspectives from McGarry, Kelly and others pointed out that abnormal fatty acid oxidation and ectopic tissue lipid accumulation are at the core of insulin resistance and T2DM genesis (see [2]; [21]), and Unger et al. have implicated lipotoxicity in pancreatic β-cell dysfunction [3]; [20]. There is now strong evidence that incomplete or inefficient LCFA β-oxidation accompanies insulin resistance and T2DM [22]; [23]. The underlying mechanisms for this association remain controversial, but it appears that sub-optimal whole-tissue tricarboxylic acid (TCA) cycle function in some tissues contributes to the metabolic phenotype in the insulin-resistant state [36]–[40]. Thus, to fully understand the metabolic landscape of T2DM, it is necessary to comprehensively determine how multiple pathways change in this condition and to evaluate how disparate pathways interconnect. Efforts to identify specific metabolites associated with T2DM and insulin resistance hold promise in developing clinically facile, predictive diagnostic and prognostic toolsets to predict diabetes risk, to determine disease stage, elucidate causal mechanisms, and to evaluate the efficacy of modalities aiming to thwart T2DM.

We have taken advantage of advancements in analytical chemistry and informatics tools to determine diabetes-associated patterns of >350 plasma metabolites encompassing carbohydrate, lipid, amino acid, purine, and organic acid classes. These results complement and extend our previous results regarding plasma acylcarnitines in the same subjects [23]. Marked differences in the plasma metabolome of diabetic and non-diabetic obese African-American women were observed, and identified a set of specific metabolites whose variability was strongly correlated with HbA1c, an index of long-term blood sugar control. These metabolites, therefore, appear to be good candidate biomarkers of blood sugar control and may provide insights into metabolic disease etiology. In at least some cases, differentially-abundant plasma metabolites appear to have resulted from hyperglycemia and increased flux of excess glucose toward secondary conversion pathways: i.e., increased fasting plasma fructose concentrations in T2DM subjects indicative of fructose generation (**Table 2**; also see [41]), and elevated glucuronic acid and xylose (**Table 2**). The concentrations of less than 10% of the measured metabolites in the current study displayed significant differences of 2-fold or greater when comparing T2DM vs. non-diabetic subjects, and these metabolites might be some of the strongest biomarkers of metabolic health status. Additional potential candidate diabetes biomarkers emerged from PCA analysis, including 93 metabolites for which variance-derived loading values were >1 standard deviation from the mean (**Table S3**) indicating their strong influence on PC scores that separated diabetics from non-diabetics (**Figure 4**). The PCA metabolite loading value patterns (**Table S3**) revealed that many metabolites reflecting the diabetic phenotype discovered here await identification since their retention times and m/z ratios did not match standards available to the investigators at the time of analysis. Furthermore, metabolite loading values in both PC1 and PC2 indicated that separation of diabetic and non-diabetic metabolic phenotypes involved variation in several chemical classes, *viz*. select carbohydrate derivatives (i.e., fructose, glucuronate, etc.), amino acids, and fatty acids had similar loading patterns in both PC dimensions (see Figure 4 legend). This indicates a concurrent impact of diabetes on intermediary metabolism of all classes of macronutrients.

Several recent investigations of urine and blood metabolomics patterns associated with T2DM or insulin resistance have provided insights into pathways influenced by these conditions (i.e., [8]; [23]; [42]–[46]). One common finding comparing our results to those of others [42]; [43]; [46] is a higher concentration of 2-hydroxybutanoic acid (2-HB; α-hydroxybutryrate) in T2DM biofluids. Plasma concentrations of 2-HB were reported to be negatively correlated with insulin sensitivity [46], and our results are consistent with this observation in that plasma 2-HB concentrations were positively correlated with HbA1c in our cohort (r = 0.455, P = 0.001). Gall et al. speculated that increased 2-HB results from higher conversion of amino acid-derived 2-ketobutanoic acid (2-KB; α-ketobutyrate) to 2-HB via lactate dehydrogenase, in conjunction with increases in the tissue NADH/NAD+ ratio concurrent with reduced insulin sensitivity and increased LCFA catabolism. Since the 2-HB precursor 2-KB is a product of cystothionine gamma-ligase activity that produces 2-KB plus cysteine, higher plasma concentrations of cysteine and its related metabolite cystine in insulin-resistant states (**Table S2**; also see [46]; [47]) support the idea that flux through the methionine/cystathionine catabolic pathway is altered with increasing insulin resistance.

Further supporting that insulin resistance and T2DM are conditions associated with abnormal amino acid metabolism, elevated blood concentrations of BCAAs have been consistently observed in rodent models of obesity [5]–[9]; [48] and in obese and/or T2DM human subjects (i.e., [8], [49]. In line with these findings, plasma leucine, 2-ketoisocaproic acid, and valine concentrations and/or their relative abundance in the total AA pool were increased in obese type 2 diabetic vs. obese non-diabetic African-American women in the current study (**Figure 2**). It has been asserted that higher concentrations of BCAA in obesity cause or exacerbate insulin resistance through mechanisms involving activation of the molecular target of rapamycin (mTOR)[8]. However, this model remains controversial. First, it is not clear that the magnitude of increase in fasting blood BCAA in obesity or T2DM are of high enough magnitude to trigger mTOR to a level that would negatively impact insulin action *in situ*. Second, BCAA-rich dairy-based diets have consistently been shown to have anti-obesity properties in rodent models (see [50]). Third, leucine supplementation to diet-induced obese mice either had no effect [51] or substantially improved [52] metabolic profiles. Finally, protein-rich diets often have positive metabolic effects in type 2 diabetic and obese humans (see [53]; [54]).

Thus, we favor the idea that higher fasting blood BCAA (and some other AA) in insulin-resistant states results simply marks reduced catabolism in key tissues, and this reduced catabolism is hypothesized to limit tissue concentrations of AA derivatives important to normal metabolism. Supporting this perspective are reports indicating that the two initial catabolic enzymes of BCAA catabolism, mitochondrial branched-chain amino acid aminotransferase (BCATm) and branched-chain α-ketoacid dehydrogenase (BCKD) expression and specific activities are reduced in the liver and/or white adipose tissue (WAT) of obese rodents [6]; [7]; [9]; [48]. She et al. [6] demonstrated that obese human subjects who underwent bariatric surgery had significantly reduced plasma BCAA concentration >1 yr post-surgery concurrent with increased WAT BCKD and BCATm activities. Whole-body BCAA clearance was reportedly ∼20% reduced in T2DM subjects [4] and recently an insulin resistance effect on leucine protein fractional synthesis rate was demonstrated [55]; [56]. However, other reports have indicated that leucine oxidation or amino acid turnover is not impaired in obese or type 2 diabetic persons [57]–[63]. Clearly, additional research is needed to identify tissue-specific alterations in the fates of protein and amino acids in obesity and diabetes to better understand the basis for elevations in blood concentrations of BCAA and certain other amino acids under these conditions. Interestingly, higher circulating concentrations of 2-HB are associated with perturbation in biotin metabolism, specifically in inherited biotinidase deficiencies [64], and poor biotin status has been associated with insulin resistant states ([65] and references therein). Biotin is crucial for proper activities of carboxylation enzymes involved in BCAA and cysteine catabolism and TCA cycle anaplerosis (i.e., propionyl-CoA carboxylase, methylcrotonyl-CoA carboxylase, pyruvate carboxylase). Thus, while speculative at this time, it is intriguing to consider that the observed higher plasma 2-HB concentrations with increasing insulin resistance marks dysfunctional biotin tissue bioactivity that would in theory impact BCAA and cysteine/cystine metabolism.

Mitochondrial dysfunction is critical in driving the diabetic phenotype. Most investigations found a reduced number or attenuated *in vivo* activity of muscle mitochondria in insulin resistant subjects [39]; [66]; [67] [36]–[38], but others have reported that the function of isolated muscle mitochondria is normal in T2DM [68]; [69]. This raises the possibility that factors limiting mitochondrial activity in diabetics *in situ* are lost in studies in isolated organelles. A notable difference is that anaplerotic carbon sources such as malate, that act to replenish TCA cycle intermediate (TCAi) carbon loss due to cataplerosis (i.e., loss of TCAi carbon via export of α-ketoglutarate or its conversion to glutamate) are invariably included in isolated mitochondrial preparations to maintain function. In a previous paper using samples from the current cohort of subjects, we observed that increased plasma concentrations of acylcarnitine and medium- and long-chain fatty acylcarnitines were coupled to reductions in propionylcarnitine concentrations in T2DM. These findings are consistent with a working model of limited tissue TCA cycle capacity relative to mitochondrial fuel delivery, due in part to sub-optimal anaplerosis pathways that replenish TCAi carbon losses from cataplerosis *in vivo* [23]. This “anaplerotic stress” model of T2DM, and the associated concept of anaplerosis/cataplerosis balance in diabetes [70] remain to be rigorously tested. There is evidence for reduced tissue concentrations of TCAi in rodent obesity and diabetes models [22]. While it is not certain if blood or urine patterns of TCAi reflect intramitochondrial patterns, in T2DM humans urinary α-ketoglutarate levels were reduced [42] and decreased urinary phenylacetyl-glutamine–a metabolite in equilibrium with glutamate and α-ketoglutarate–has been reported in T2DM [45]. It is tempting to consider that dysfunctional BCAA and cysteine catabolism (see discussion above) contribute to anaplerotic stress associated with insulin resistance, since valine, isoleucine, and cysteine are precursors to succinate and succinyl-CoA. Consistent with this view, plasma valine enrichment in the amino acid pool rose concurrent with reductions in relative concentrations of propionylcarnitine (a proxy for a valine anaplerotic product, propionyl-CoA) as blood sugar control worsened and plasma acetylcarnitine accumulation became more apparent in our study cohort **(** **Figure 5** **)**. In theory, changes in matrix α-ketoglutarate concentration (i.e., via conversion to glutamate or export via the α-ketoglutarate carrier) could impact both TCA cycle function and BCAA metabolism, since BCATm activity involves α-ketoglutarate. Notably, Seifert et al. recently reported that increases in the rate of LCFA combustion and concurrent incomplete LCFA β-oxidation are associated with muscle mitochondrial export of α-ketoglutarate [71], providing a potential connection between mitochondrial lipid metabolism and control of α-ketoglutarate export. The idea that anaplerotic stress and anaplerotic/cataplerotic balance are factors underlying or exacerbating metabolic dysfunction in T2DM is compelling, but clearly requires experimental validation.

Our work has been driven in part by a desire to identify muscle-specific metabolites associated with LCFA β-oxidation, considering the importance of efficient skeletal muscle LCFA catabolism to maintenance of insulin sensitivity. An interesting group to study in this regard are non-diabetic obese African-American persons harboring a G304A (g/a) missense allele leading to truncated UCP3, and who have been found to have significantly reduced whole-body lipid oxidation [27]. This must in large part emanate from muscle considering the almost exclusive expression of this mitochondrial carrier in that tissue in humans 72. Thus, differences in circulating metabolites when comparing g/g to g/a individuals may reflect differences in muscle metabolism. In this study, we found several metabolites that were altered in overnight-fasted non-diabetic g/a allele carriers (**Table 1**, **Table S1**), and most await identification and to ascertain tissue-specificity of their production and utilization. Many metabolite differences between genotypes were statistically significant, but significance was not detected after application of a 20% false discovery rate. This was likely due to limited sample sizes, and indicates that additional comparative studies are warranted using larger cohorts of g/g and g/a polymorphic subjects to confirm metabolite differences observed herein. We observed reductions in plasma concentrations of α-ketoglutarate and glutamate in g/a individuals, as well as lower cysteine, suggesting that alteration in UCP3 function in muscle of obese African-American women impacts TCAi dynamics and amino acid metabolism. Plasma butyrylcarnitine (C4-carnitine) was previously found to be reduced by 57% in the same non-diabetic g/a subjects [23]. Plasma butyrylcarnitine should be reflective of tissue butyryl-CoA concentrations, and importantly the latter is a common metabolite in the catabolism of both glutamate and α-ketoglutarate. Thus, we propose from the aggregate of results that muscle UCP3 activity may somehow play a role in regulating tissue α-ketoglutarate/glutamate dynamics.

In summary, broad metabolite profiling of non-diabetic vs. type 2 diabetic plasma, in conjunction with our previous acylcarnitine profiling, indicates that T2DM is a disease that disrupts multiple intermediary metabolic pathways including amino acid metabolism. Our data support a hypothetical working model in which attenuated BCAA, and possibly cysteine, catabolism contribute to increased blood concentrations of these amino acids and their derivatives in the insulin-resistant state, and we speculate that this contributes to anaplerotic stress that is associated with incomplete oxidation of LCFA and accumulation of acylcarnitines in T2DM. Variation in a subset of metabolites, including 2-HB, discriminated T2DM from non-diabetics and strongly correlated with HbA1c. These metabolites therefore are of interest as potential biomarkers of disease status and glucose homeostasis. Our results also point to, for the first time, a potential role of mitochondrial UCP3 in regulation of the glutamate/α-ketoglutarate/butanoyl-CoA tissue pool.

## Supporting Information

Table S1 **Plasma Metabolite list comparing UCP3 g/g and g/a polymorphs in an obese, African-American cohort of women.** Values are quantifier peak heights determined for each subject used in the analyses, and metabolites are identified in the leftward column. Mass spec information is provided in columns B–E, and in some cases, KEGG identifiers are provided. Unpaired t-tests were used to evaluate non-diabetic genotype differences, as noted.
(XLS)

Table S2 **Plasma Metabolite list comparing type 2 diabetic and non-diabetic obese African-American women.** Values are quantifier peak heights determined for each subject used in the analyses, and metabolites are identified in the leftward column. Mass spec information is provided in columns B–E, and in some cases, KEGG identifiers are provided. Unpaired t-tests were used to evaluate differences between diabetic and non-diabetic women, as noted.
(XLS)

Table S3 **Loadings values for each of the principal components (metabolite factors) used to generate subject PC scores depicted in** **Figure 4** **.**
(XLS)
